# Delayed Emergence Due to Prolonged Mivacurium Blockade in a Child with Markedly Reduced Butyrylcholinesterase Activity: A Case Report

**DOI:** 10.3390/clinpract16100186

**Published:** 2026-10-09

**Authors:** Daniela Saburova, Rihards Peteris Rocans, Irina Evansa, Arta Barzdina, Inna Babuskina, Natalija Zlobina, Olegs Sabelnikovs

**Affiliations:** 1 Department of Anaesthesia and Intensive Care, Riga Stradiņš University, LV-1007 Riga, Latvia; rihardspeteris.rocans@rsu.lv (R.P.R.); irina.evansa@1slimnica.lv (I.E.); arta.barzdina@rsu.lv (A.B.); olegs.sabelnikovs@rsu.lv (O.S.); 2 Department of Anaesthesiology, Intensive Care and Pain Management, Riga 1st Hospital, LV-1001 Riga, Latvia; natalija.zlobina@1slimnica.lv; 3 Vītola Clinic, LV-1045 Riga, Latvia; inna.babuskina@gmail.com; 4 Department of Intensive Care, Children’s Clinical University Hospital, LV-1004 Riga, Latvia

**Keywords:** delayed emergence, pediatric anesthesia, mivacurium, butyrylcholinesterase deficiency, residual neuromuscular blockade

## Abstract

**Background:** Delayed emergence after general anesthesia in pediatric patients is uncommon and requires prompt evaluation of pharmacological, metabolic, and neurological causes. Residual neuromuscular blockade remains an important reversible cause, particularly when short acting neuromuscular blocking agents dependent on plasma cholinesterase metabolism are used. **Case report:** We report the case of a previously healthy three-year-old boy who underwent elective adenoidectomy, tonsillotomy, and tympanostomy under general anesthesia with fentanyl, propofol, sevoflurane, and mivacurium. Following the procedure, the patient failed to regain spontaneous breathing, purposeful movements, or response to painful stimuli. In response, neostigmine was administered to reverse possible residual neuromuscular blockade; however, no immediate clinical improvement was observed, and mechanical ventilation was continued. Quantitative neuromuscular monitoring was unavailable. Therefore, residual blockade could not be objectively assessed, and the depth of blockade at the time of neostigmine administration remained unknown. The patient was transferred to a tertiary pediatric center for further evaluation. Several hours later, spontaneous breathing and motor activity recovered, and the patient was successfully extubated. Retrospective family history revealed prolonged recovery after anesthesia in the patient’s grandfather. Subsequent laboratory testing demonstrated markedly reduced plasma butyrylcholinesterase activity (2493 U/L; reference range 7000–19,000 U/L), raising a strong suspicion of inherited butyrylcholinesterase deficiency. **Conclusions:** This case highlights the importance of considering residual neuromuscular blockade in delayed emergence after pediatric anesthesia, maintaining airway protection, and obtaining relevant family history to guide future management.

## 1. Introduction

Delayed emergence (DE) after general anesthesia is relatively uncommon and requires prompt evaluation [1]. DE is defined as the inability to regain an adequate level of consciousness, with the patient remaining unresponsive or deeply sedated 30–60 min after the end of general anesthesia [2]. DE after general anesthesia may result from residual anesthetic effects, metabolic disturbances, neurologic events, and respiratory causes [2,3].

Mivacurium is a short acting nondepolarizing neuromuscular blocking agent metabolized by plasma butyrylcholinesterase (also known as pseudocholinesterase) [4,5]. Reduced BChE activity may be inherited or acquired and can markedly prolong mivacurium-induced neuromuscular blockade. In a large multi-ethnic pharmacogenetic cohort of 13,301 individuals, 0.06% were genetically predicted to have severe BChE deficiency [6]. Individuals with inherited BChE deficiency are generally asymptomatic until exposed to agents metabolized by this enzyme, such as mivacurium or succinylcholine [6,7].

Although population-level pediatric data are limited, recent pediatric reports have documented prolonged mivacurium-induced neuromuscular blockade requiring postoperative ventilatory support in otherwise healthy young children with previously unrecognized BChE deficiency [7,8].

We present a case of delayed emergence in a previously healthy child following mivacurium administration during routine ENT surgery. The case highlights the diagnostic and management challenges associated with unexpected prolonged paralysis, particularly when quantitative neuromuscular monitoring is unavailable.

## 2. Case Report

A three-year-old boy weighing 15.5 kg and measuring 98 cm was scheduled for elective adenoidectomy, tonsillotomy, and tympanostomy due to adenoid hypertrophy, tonsillar hypertrophy, and secretory otitis media. The presenting symptoms included persistent mouth breathing attributed to adenoid hypertrophy and changes in hearing. No chronic inflammatory disease or history of chronic or recurrent tonsillitis was documented in the available medical records.

Preoperative assessment was unremarkable. The child had normal development, no known allergies, no chronic illnesses, and no prior anesthetic exposure. During the preoperative assessment, no known family history of anesthesia-related complications was reported. Daily medication included only vitamin D. Airway examination revealed Mallampati class I. He was classified as ASA physical status I. Baseline vital signs included heart rate 103 beats/min, oxygen saturation 98%, and blood pressure 115/72 mmHg.

One hour before surgery, the patient received oral midazolam 1.85 mg (0.12 mg/kg) as premedication.

General anesthesia was induced with fentanyl 30 µg (1.9 µg/kg), propofol 50 mg (3.2 mg/kg), and mivacurium 4 mg (0.26 mg/kg). Mask ventilation was uncomplicated, followed by atraumatic endotracheal intubation using a 4.0 mm tube.

Anesthesia was maintained with sevoflurane 1.9% in an oxygen–air mixture. Standard intraoperative monitoring included continuous electrocardiography, non-invasive blood pressure measurement, pulse oximetry, capnography, and monitoring of respiratory and anesthetic gas parameters. Mechanical ventilation was provided using SIMV PCV-VG and remained stable throughout the procedure, with oxygen saturation of 100% and end-tidal CO_2_ of approximately 40 mmHg.

The surgical procedure was uneventful. Intraoperatively, the patient received paracetamol 250 mg, metamizole 250 mg, dexamethasone 2 mg, ondansetron 2 mg, clonidine 15 µg, and 250 mL isotonic saline. A transient hypotensive episode occurred, with blood pressure decreasing to 64/38 mmHg, which responded to ephedrine 3 mg (0.19 mg/kg).

At the end of surgery, the child failed to regain spontaneous breathing, spontaneous movements, or response to painful stimuli. Quantitative neuromuscular monitoring data were unavailable. Because persistent residual neuromuscular blockade was considered among the leading differential diagnoses, neostigmine 800 µg (0.05 mg/kg) was administered at 09:35, 55 min after mivacurium administration, to reverse a possible nondepolarizing neuromuscular block. No immediate clinical improvement was observed following administration. Capillary blood glucose was 8.5 mmol/L. The patient was transferred intubated to the intensive care unit for further management.

Due to persistent absence of spontaneous breathing and motor response, an additional dose of neostigmine 250 µg (0.016 mg/kg) was administered at 11:00, 140 min after mivacurium administration. The repeated dose was administered with the intention of reversing the residual effect of the neuromuscular blocking agent. No spontaneous breathing, purposeful movement, or response to painful stimulation was observed following the second dose. Mechanical ventilation and close monitoring were continued. Before re-sedation, the patient remained hemodynamically stable, with a blood pressure of 99/60 mmHg and a heart rate of 100 beats/min. No marked tachycardia or hypertension was observed. Sedation with propofol was administered to ensure patient comfort and prevent awareness during suspected prolonged neuromuscular blockade while mechanical ventilation was maintained.

Given the lack of recovery, consultation with a tertiary pediatric center was arranged, and interhospital transfer was initiated. The patient was transported under the care of a specialized medical transport team.

**Figure 1:**
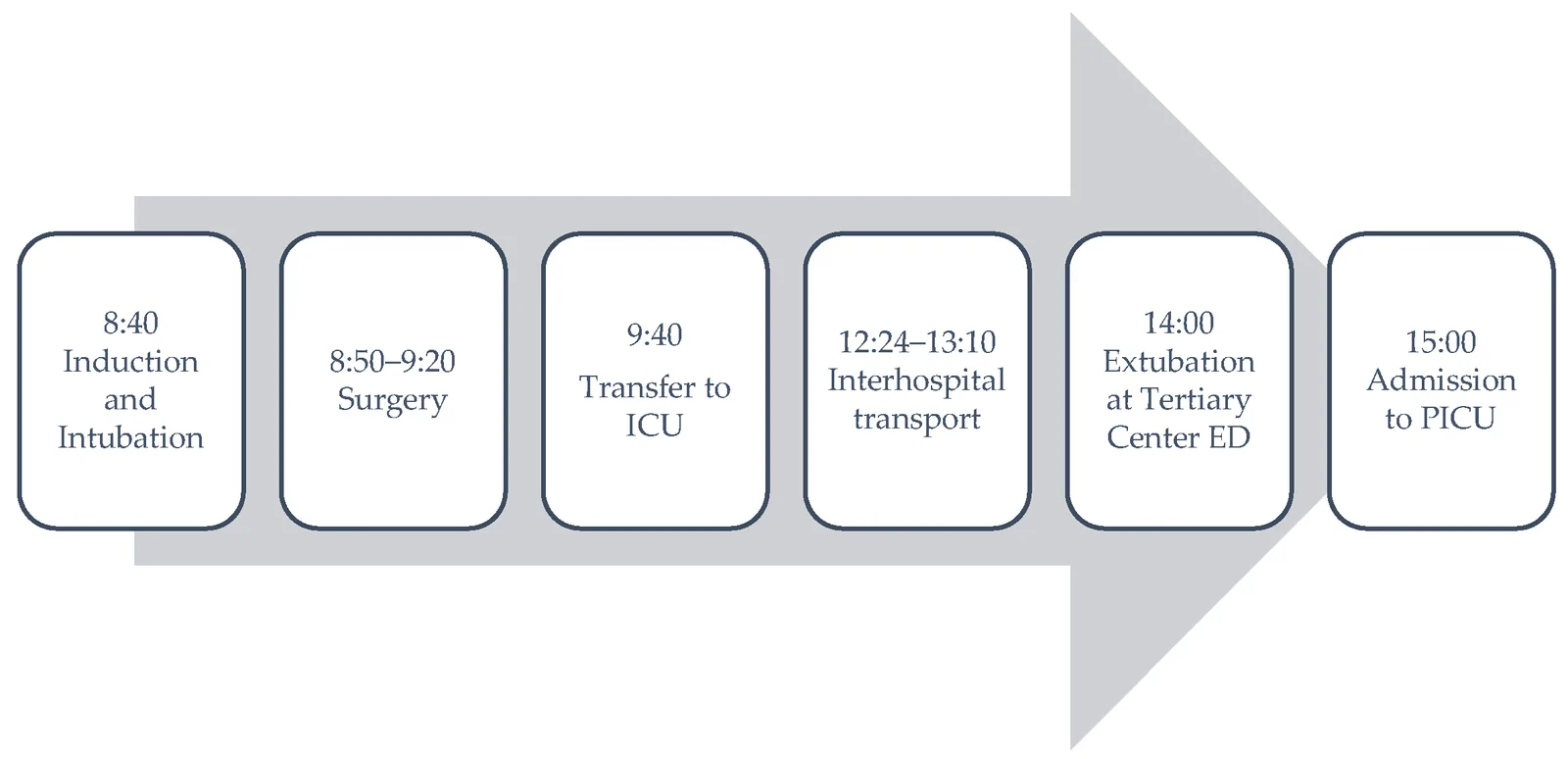
Timeline of the patient’s perioperative course and subsequent clinical recovery.

Upon arrival at the emergency department of the tertiary center, the child demonstrated signs of recovery, including spontaneous breathing, active movements, and resistance to the endotracheal tube. At approximately 14:00, the patient was successfully extubated, approximately five hours after the end of surgery. The timeline of the perioperative course, prolonged neuromuscular blockade, and subsequent recovery is summarized in Figure 1. Following extubation, he cried appropriately and requested food, indicating adequate neurological recovery. A mild transient anisocoria was noted, pupils were reactive to light and no other neurological abnormalities were observed.

At approximately 15:00, the patient was admitted to the pediatric intensive care unit (PICU) for further observation. No signs of neurological deficit were observed after recovery.

The patient remained clinically stable, spent two days in intensive care observation, and was subsequently transferred to the pediatric ward. He was discharged home in good clinical condition without apparent complications.

Following discharge, biochemical evaluation of plasma butyrylcholinesterase activity was performed because of the prolonged neuromuscular blockade and suggestive family history. Plasma butyrylcholinesterase activity was markedly reduced at 2493 U/L (reference range 7000–19,000 U/L), supporting the diagnosis of butyrylcholinesterase deficiency. Genetic testing was not performed.

Further review of the family history revealed that the patient’s grandparent had previously experienced prolonged recovery following general anesthesia. However, no biochemical or genetic testing for butyrylcholinesterase deficiency had been performed in this relative, and no confirmed diagnosis was available.

## 3. Discussion

This case illustrates an uncommon but clinically significant cause of delayed emergence in pediatric anesthesia. The main differential diagnoses included residual sedative or anesthetic drug effect, metabolic derangement, neurological pathology, and residual neuromuscular blockade [2,9]. The absence of spontaneous breathing and movement, followed by complete recovery several hours later without neurological deficit, is highly suggestive of prolonged pharmacologic paralysis, although objective confirmation with quantitative neuromuscular monitoring was unavailable. Residual neuromuscular blockade may present with apnea, respiratory insufficiency, inability to maintain airway patency, and profound weakness that can be mistaken for delayed awakening or neurological injury [8,10].

Delayed emergence in pediatric patients represents a time-critical diagnostic challenge requiring simultaneous evaluation of pharmacological, metabolic, respiratory, and neurological causes. In the absence of quantitative neuromuscular monitoring, residual neuromuscular blockade cannot be objectively confirmed or excluded during the acute phase. Consequently, clinical decision-making must rely on serial clinical assessment while maintaining airway protection and adequate ventilatory support until the underlying cause has been clarified. In our patient, this approach enabled safe transfer to a tertiary pediatric intensive care unit under controlled mechanical ventilation while continued diagnostic reassessment was undertaken [2,3,11,12].

Residual sedative or opioid effects were unlikely to fully explain the absence of spontaneous breathing and motor response. Metabolic causes were excluded based on normal glucose levels and stable intraoperative parameters, while neurological complications became less likely following complete neurological recovery [2].

Residual neuromuscular blockade may mimic coma or severe neurological deterioration, potentially leading to unnecessary neurological investigations and delays in diagnosis [5]. In this setting, premature extubation or assumptions that delayed emergence is solely due to residual anesthetic drugs may place patients at risk of airway obstruction, hypoventilation, and hypoxemia. Until the underlying cause has been clarified, maintaining mechanical ventilation and close clinical observation remain the safest management strategy.

Mivacurium is hydrolyzed by plasma butyrylcholinesterase, and inherited deficiency of this enzyme may result in markedly prolonged neuromuscular blockade. In children, butyrylcholinesterase activity is generally higher than in adults, and mivacurium is characterized by a faster onset and shorter duration of action [5]. Therefore, unexpectedly prolonged paralysis following mivacurium administration should raise suspicion of reduced or abnormal butyrylcholinesterase activity [5]. The expected clinical duration of mivacurium following an intubating dose is approximately 15–20 min. However, recovery in our patient occurred approximately 5 h after administration. Together with the markedly reduced plasma butyrylcholinesterase activity and positive family history, these findings are highly suggestive of inherited butyrylcholinesterase deficiency [5]. Similar recovery times ranging from four to six hours have been reported in pediatric patients with previously undiagnosed butyrylcholinesterase deficiency [7,8].

In the present case, plasma butyrylcholinesterase activity was markedly reduced (2493 U/L; reference range 7000–19,000 U/L). However, confirmatory genetic testing or biochemical phenotyping was not performed [5,13].

Reduced plasma butyrylcholinesterase activity may result from either inherited or acquired conditions. Reported acquired causes include liver disease, malnutrition, chronic inflammatory states, renal disease, malignancy, severe burns, and treatment with certain medications [5,13,14]. Although these conditions cannot be completely excluded, the patient’s unremarkable medical history, young age, and suggestive family history make an inherited etiology the most likely explanation.

The role of neostigmine in mivacurium-induced neuromuscular blockade is complex and depends on the depth of blockade at the time of reversal. Neostigmine increases acetylcholine availability at the neuromuscular junction and can therefore antagonize nondepolarizing neuromuscular blockade; however, it also inhibits plasma butyrylcholinesterase, the principal enzyme responsible for mivacurium hydrolysis [5]. A systematic review found that neostigmine modestly accelerated recovery when administered during moderate mivacurium blockade, whereas its effect during deep blockade remains uncertain [15]. This creates a clinically relevant dilemma when prolonged mivacurium effect is suspected, particularly in patients with reduced butyrylcholinesterase activity. In our patient, quantitative neuromuscular monitoring data were unavailable; therefore, the depth of blockade and degree of spontaneous recovery at the time of neostigmine administration could not be established. Consequently, the effect of neostigmine on the subsequent clinical course cannot be determined retrospectively. This case highlights the importance of quantitative neuromuscular monitoring to guide both the timing and appropriateness of pharmacological reversal.

Unfortunately, train-of-four monitoring data were not available. Therefore, prolonged residual neuromuscular blockade could not be objectively confirmed and remains the most likely explanation based on the clinical course, markedly reduced butyrylcholinesterase activity, and suggestive family history. Routine quantitative neuromuscular monitoring facilitates earlier recognition of residual neuromuscular blockade, improves diagnostic confidence, guides appropriate reversal strategies, and supports safer extubation decisions [11,12]. When quantitative monitoring is unavailable, qualitative assessment using a peripheral nerve stimulator may still provide clinically useful information regarding the presence of neuromuscular blockade and evidence of spontaneous recovery; however, it cannot reliably exclude residual neuromuscular blockade or confirm adequate recovery [11,16]. In the present case, quantitative monitoring could have distinguished persistent neuromuscular blockade from other causes of delayed emergence, objectively assessed the depth of blockade and spontaneous recovery, and guided the timing and appropriateness of neostigmine administration. Serial measurements could also have documented recovery from the prolonged block and provided objective evidence to support safe extubation.

Although family history of anesthesia-related complications was specifically assessed preoperatively, no relevant history was known to the family at that time. The grandparent’s prolonged recovery from general anesthesia was identified only after the present event through further discussion with relatives. This illustrates that a negative preoperative family history does not necessarily exclude an inherited susceptibility when family members are unaware of previous anesthesia-related events.

An awake but paralyzed patient may exhibit indirect signs of distress or sympathetic activation, such as tachycardia or hypertension; however, these findings are nonspecific and their absence does not exclude awareness [17]. In the present case, the patient remained hemodynamically stable before re-sedation, without marked tachycardia or hypertension. Therefore, the vital signs alone did not allow reliable differentiation between persistent neuromuscular blockade and other causes of delayed emergence.

Several pediatric cases of prolonged mivacurium-induced neuromuscular blockade associated with butyrylcholinesterase deficiency have recently been reported [7,8]. However, unlike recently published cases in which the diagnosis was confirmed using quantitative neuromuscular monitoring together with biochemical and/or genetic investigations, the present case reflects a real-world clinical scenario in which objective confirmation of residual neuromuscular blockade was not available during the acute phase. Therefore, the clinical value of this report lies not in the rarity of the diagnosis itself, but in highlighting the importance of a structured differential diagnostic approach, early supportive management, and patient safety, while the diagnosis remains uncertain [11,12].

A limitation of this report is the absence of confirmatory testing for inherited butyrylcholinesterase deficiency. Although plasma butyrylcholinesterase activity was markedly reduced, genetic testing, biochemical phenotyping (e.g., dibucaine number determination), and repeat measurement of enzyme activity were not performed. Therefore, inherited butyrylcholinesterase deficiency could not be definitively confirmed. In addition, acquired causes of reduced butyrylcholinesterase activity, including liver disease, malnutrition, chronic inflammatory conditions, and certain medications, could not be completely excluded. However, the absence of known risk factors for acquired butyrylcholinesterase deficiency together with the suggestive family history make an inherited etiology the most likely explanation. Current evidence suggests that combining biochemical phenotyping with molecular testing provides more accurate characterization of butyrylcholinesterase deficiency and facilitates family counselling [5,18,19].

## 4. Conclusions

Residual neuromuscular blockade should be considered in pediatric patients with unexpected delayed emergence after anesthesia when neuromuscular blocking agents have been used. Early airway protection, continued ventilatory support, careful clinical reassessment, and a structured differential diagnostic approach are essential for patient safety, particularly when quantitative neuromuscular monitoring is unavailable.

## Data Availability

The original contributions presented in this study are included in the article. Further inquiries can be directed to the corresponding author.

## Abbreviations

The following abbreviations are used in this manuscript:

ASA	American Society of Anesthesiologists
DE	Delayed emergence
ENT	Ear, nose and throat
ICU	Intensive care unit
PICU	Pediatric intensive care unit
ED	Emergency department
